# Radiomics Analysis on Multiphase Contrast-Enhanced CT: A Survival Prediction Tool in Patients With Hepatocellular Carcinoma Undergoing Transarterial Chemoembolization

**DOI:** 10.3389/fonc.2020.01196

**Published:** 2020-07-21

**Authors:** Xiang-Pan Meng, Yuan-Cheng Wang, Shenghong Ju, Chun-Qiang Lu, Bin-Yan Zhong, Cai-Fang Ni, Qi Zhang, Qian Yu, Jian Xu, JianSong Ji, Xiu-Ming Zhang, Tian-Yu Tang, Guanyu Yang, Ziteng Zhao

**Affiliations:** ^1^Jiangsu Key Laboratory of Molecular and Functional Imaging, Department of Radiology, Zhongda Hospital, Medical School of Southeast University, Nanjing, China; ^2^Department of Interventional Radiology, The First Affiliated Hospital of Soochow University, Suzhou, China; ^3^Department of Interventional Radiology, Jinling Hospital, Medical School of Nanjing University, Nanjing, China; ^4^Department of Radiology, Affiliated Lishui Hospital of Zhejiang University, The Central Hospital of Zhejiang Lishui, Lishui, China; ^5^Department of Radiology, Nanjing Medical University Affiliated Cancer Hospital, Cancer Institute of Jiangsu Province, Nanjing, China; ^6^LIST, Key Laboratory of Computer Network and Information Integration, Southeast University, Ministry of Education, Nanjing, China

**Keywords:** hepatocellular carcinomas, image processing (computer-assisted), radiomics, transarterial chemoembolization, biomarkers

## Abstract

Patients with HCC receiving TACE have various clinical outcomes. Several prognostic models have been proposed to predict clinical outcomes for patients with hepatocellular carcinomas (HCC) undergoing transarterial chemoembolization (TACE), but establishing an accurate prognostic model remains necessary. We aimed to develop a radiomics signature from pretreatment CT to establish a combined radiomics-clinic (CRC) model to predict survival for these patients. We compared this CRC model to the existing prognostic models in predicting patient survival. This retrospective study included multicenter data from 162 treatment-naïve patients with unresectable HCC undergoing TACE as an initial treatment from January 2007 and March 2017. We randomly allocated patients to a training cohort (*n* = 108) and a testing cohort (*n* = 54). Radiomics features were extracted from intra- and peritumoral regions on both the arterial phase and portal venous phase CT images. A radiomics signature (Rad-signature) for survival was constructed using the least absolute shrinkage and selection operator method in the training cohort. We used univariate and multivariate Cox regressions to identify associations between the Rad- signature and clinical factors of survival. From these, a CRC model was developed, validated, and further compared with previously published prognostic models including four-and-seven criteria, six-and-twelve score, hepatoma arterial-embolization prognostic scores, and albumin-bilirubin grade. The CRC model incorporated two variables: The Rad-signature (composed of features extracted from intra- and peritumoral regions on the arterial phase and portal venous phase) and tumor number. The CRC model performed better than the other seven well-recognized prognostic models, with concordance indices of 0.73 [95% confidence interval (CI) 0.68–0.79] and 0.70 [95% CI 0.62–0.82] in the training and testing cohorts, respectively. Among the seven models tested, the six-and-12 score and four-and-seven criteria performed better than the other models, with C-indices of 0.64 [95% CI 0.58–0.70] and 0.65 [95% CI 0.55–0.75] in the testing cohort, respectively. The CT radiomics signature represents an independent biomarker of survival in patients with HCC undergoing TACE, and the CRC model displayed improved predictive performance.

## Introduction

Several treatment guidelines recognize that transarterial chemoembolization (TACE) brings significant survival benefit over supportive care in patients first diagnosed with Barcelona Clinic Liver Cancer (BCLC) stage B hepatocellular carcinomas (HCC) (1–3). Despite receiving similar treatment, these patients experienced substantial survival heterogeneity after TACE (2), rendering the building of risk stratification algorithms essential. Several existing prognostic models, including the four-and-seven criteria, six-and-12 score, hepatoma arterial-embolization prognostic (HAP) scores, and albumin-bilirubin grade, have been proposed to predict clinical outcome after TACE (4–7). Some of cohort studies also indicated there is space for prognostic accuracy improvement (8, 9). Developing biomarkers from routinely collected data into an improved prognostic model will help identify optimal candidates for TACE.

Computed tomography (CT) imaging has a fundamental role in the diagnosis, staging, treatment guidance, and response monitoring in HCC (10). Indeed, CT images of HCC also provide quantifiable and non-invasive imaging biomarkers for prognostics, including comprehensive information on the shape, intensity, and enhancement of the entire tumor (11, 12). According to the modified Response Evaluation Criteria in Solid Tumors (mRECIST) criteria or the European Association for the Study of the Liver (EASL) criteria (3, 13), axial tumor size was routinely used to categorize tumor response. However, this measurement is subject to interobserver variability and inherently inexact compared to assessing 3D tumor volume (14, 15). While a few reports have proposed qualitative imaging traits (“tumor capsule” or “internal arteries”) as potential predictors, these remain highly dependent on radiologists' experience (16, 17). Thus, a novel and precise method of comprehensively quantifying the pretreatment CT information is urgently needed to identify non-invasive biomarkers.

Radiomics, an emerging approach that converts medical images into high-dimensional quantifiable data, has exhibited increasing prognostic power by capturing distinct phenotypic differences of tumors (18). A few studies reported that texture analysis on arterial phase CT imaging predicted therapeutic response and survival in patients with HCC after TACE (19, 20). However, applying radiomics on multiphasic contrast-enhanced CT imaging to predict survival after TACE is rarely investigated. Some studies demonstrated that analyzing the texture of both the intratumoral plus peritumoral regions provided superior prognosis prediction for patients with HCC compared to the intratumoral region alone (21, 22). Therefore, we hypothesized that a radiomics pattern from peritumoral regions might be valuable for prognosis prediction.

Therefore, this study aimed to improve the current survival prediction models for patients with HCC through the following: (1) building a radiomics signature integrating both intratumoral and peritumoral CT radiomics patterns; (2) developing and validating a combined radiomics-clinic (CRC) model; (3) and comparing the ability of the CRC model and existing prognostic models to predict survival.

## Materials and Methods

### Patients and Study Design

This study was approved by the Institutional Review Board and the need to obtain informed consent was waived because of the retrospective nature of the study.

We retrospectively identified 911 consecutive patients with HCC who underwent TACE between January 2007 and March 2017 as the first-line therapy at five centers in China. HCC was diagnosed histologically or by CT image evaluation, according to the European Association for the Study of the Liver or American Association for the Study of Liver Diseases criteria. The inclusion criteria included: (1) patients with HCC receiving TACE as initial treatment who had (2) complete clinical data. Patients were excluded based on the following criteria: (1) Missing or inadequate baseline contrast-enhanced CT imaging within 6 weeks before treatment initiation (*n* = 617); (2) Infiltrative disease (*n* = 7); (3) Eastern Cooperative Oncology Group (ECOG) performance status score > 0 (*n* = 17); (4) Child-Pugh classification C or D (*n* = 8); (5) Presence of macrovascular invasion or extrahepatic metastasis (*n* = 166). Notably, criteria 3–5 excluded BCLC stage C patients, for which TACE is much less effective (2). Finally, we included the patients at BCLC stage B (*n* = 154) and BCLC stage A (*n* = 8) carefully defined as unresectable due to tumor location or patient status. For independent validation, we allocated patients who first underwent TACE before May 2014 to a training cohort (*n* = 108), and subsequent patients were allocated to a testing cohort (*n* = 54). Similar to previous study (5), we did not split data by center (external validation) (23).

### TACE Procedure

TACE was administered using mixtures of lipiodol and chemotherapeutic drugs (pirarubicin, cisplatin, or epirubicin were selected according to the practice of each center), followed by embolization using a gelatin sponge. Either selective or super-selective embolization of the tumor-feeding vessels was performed whenever technically reasonable (24). The dose of lipiodol and chemotherapeutic drugs was based on tumor burden and patients' characteristics. Investigators with at least 8 years of experience performed all procedures. When no vital tumor tissue was observed on contrast-enhanced CT or magnetic resonance imaging (MRI) 4–6 weeks after initial TACE treatment, TACE was discontinued. “On-demand” TACE procedures were repeated at an interval of 6–12 weeks in patients with viable tumors or intrahepatic recurrences observed by contrast-enhanced CT/MRI but without extrahepatic spread or deterioration in clinical status (25).

### Image Acquisition Parameters

All patients underwent multiphasic contrast-enhanced abdominal CT scan using one of the following systems: Discovery CT750 HD (GE Medical System), LightSpeed VCT (GE Medical System), iCT 128 (Philips), iCT 256 (Philips), Mx8000 (Philips), Sensation 64 CT (Siemens), Somatom Definition (Siemens), or Toshiba (Aquilion). Scanning parameters are as follows: 120–140 kVp; 150–190 mAs; field of view, 350 × 350 mm; matrix, 512 × 512. Table S1 details the parameters of slice thickness and pixel spacing. A 1.5–2.0 mL/kg body weight bolus of contrast material iodixanol (Ultravist 370, Bayer, Germany) was injected intravenously at a flow rate of 3–4.0 mL/sec. Arterial phase, portal venous phase, and equilibrium phase were performed with bolus triggering, typically ~30, 60–70, and 180 s, respectively, after injection of contrast. We retrieved the arterial phase and portal venous phase images from the picture archiving and communication system of the five centers and downloaded images in a Digital Imaging and Communications in Medicine format.

### Volume of Interest Segmentation and Radiomics Feature Extraction

The volume of interest (VOI) included both tumor and peritumoral regions. Firstly, a radiologist (reader 1, XM, a radiologist with 6-years abdominal imaging experience) manually annotated 3D tumor VOIs around the largest lesion on both arterial and portal venous phase images using ITK-SNAP version 3.6 (http://www.itksnap.org). To evaluate the reproducibility of the extracted features, reader 2 (QY, a radiologist with 5-years abdominal imaging experience) independently segmented randomly selected 50 lesions from both arterial and portal venous phase CT scans. The intraclass correlation coefficient (ICC) was used to validate the reproducibility of extracted features from the two radiologists. Only features with an inter-reader ICC > 0.75 were included in subsequent analyses. After the tumor VOI was segmented, we considered the pixel size of each CT scan to perform a morphologic dilation operation, capturing the peritumoral region of the entire tumor VOI, with a radial distance of 10 mm. A peritumoral VOI of the liver parenchyma immediately surrounding the tumor was obtained after subtracting the tumor VOI from this dilated VOI. Appendix E1 provides further details on generating tumor segmentation and peritumoral VOI.

Radiomics features were extracted from each VOI by using Pyradiomics 2.0.0 (https://pyradiomics.readthedocs.io/en/latest/features.html) (26). Images were isotopically resampled to 1 × 1 × 1 mm^3^ voxels with a fixed bin width of 25 for image discretization. Detailed descriptions are provided under the “Imaging preprocessing” in Appendix E2. For each VOI, we extracted a radiomics set of 1,288 features comprised of four categories (Appendix E2): shape features (*n* = 14), the first-order features (*n* = 18), the second-order features (*n* = 23), and high-order filters features (generated by Laplacian of Gaussian filter and wavelet filter, *n* = 1,183 features). For each lesion, we extracted 5,152 radiomics features from tumor and peritumoral VOI in both the arterial phase and portal venous phase images. All feature extraction methods conformed to the image Biomarkers Standardization Initiative (IBSI) guidelines (27). Feature *Z*-score normalization was performed first in the training cohort. The testing cohort was *Z*-score normalized using the training cohort as a “reference;” the mean and standard deviation values used to *z*-score normalize the feature values in the testing cohort were identical in the training cohort.

### Radiomics Feature Selection and Signature Building

**Figure 1:**
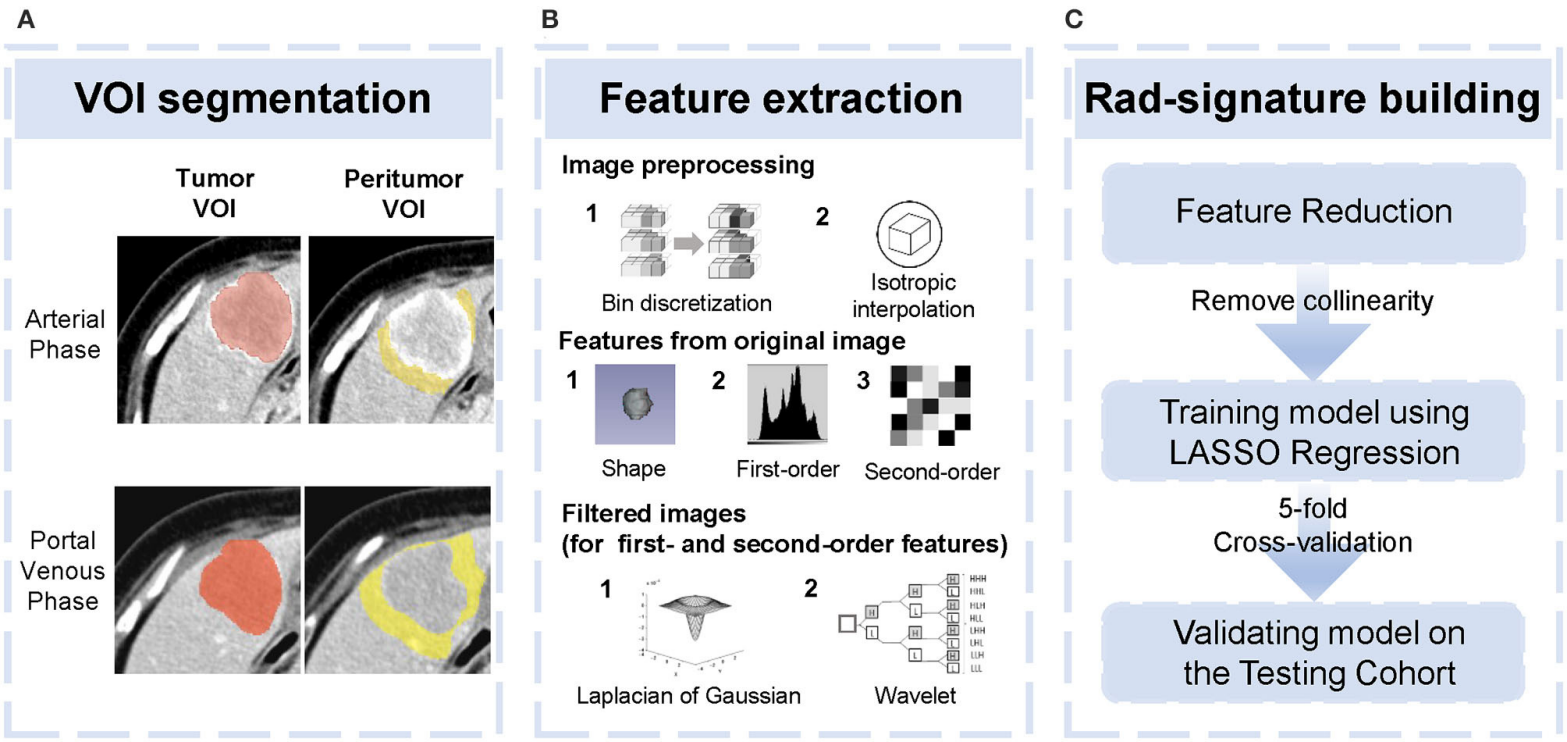
Overview of radiomics analysis in this study. **(A)** tumor volume of interest (VOI) and peritumoral VOI segmentation. **(B)** Image pre-processing and feature extraction from original and filtered images. **(C)** Feature reduction and development and validation of the Rad-signature.

Firstly, pair-wise correlations analysis was performed to remove redundant radiomics features, by using the “findCorrelation” function in R package “caret” with the absolute correlation cutoff set at 0.9. Then, we employed the least absolute shrinkage and selection (LASSO) Cox regression (28), a qualified approach for regression of high-dimensional predictors by a penalty to shrink some regression coefficients to exactly zero. This approach selected the most predictive radiomics features from the training cohort. The penalty parameter (lambda) was determined by using 5-fold cross-validation based on minimum error criteria. Selected features were weighted by their respective coefficients obtained from LASSO, and we computed a radiomics signature (Rad-signature) with a linear combination of these features. Identical coefficient values were applied to the testing cohort. An overview of radiomics analysis is shown in Figure 1.

### Statistical Analysis

Continuous variables are reported as median (interquartile range [IQR]) and were compared using the Mann-Whitney *U*-test, whereas all categorical variables were summarized as number (percent) and compared using the Fisher's exact test. Survival curves were depicted using the Kaplan-Meier method and compared by the log-rank test. Overall Survival (OS) was defined as the time interval between initial TACE and all-cause death. Data concerning patients who were lost to follow-up or survived at the last follow-up (November 16, 2018) were censored.

Univariate Cox regression analyses were used to ascertain prognostic clinical factors. A potential correlation was regarded as present if *P* ≤ 0.1. With multivariate Cox regression analyses, a combined radiomics-clinic (CRC) model was developed using the Rad-signature and clinical factors with a potential association with OS. Final model selections were performed by stepwise backward selection with the Akaike information criterion. Consistent with previously well-recognized studies, we treated alpha-fetoprotein (AFP) (>400 vs. ≤400 ng/mL) as a binary variable in regressions. A radiologist (YW, with 15-years abdominal imaging experience) who was blinded to the clinical data of patients evaluated the diameter of the largest nodule (tumor size) and tumor number. Because of sparse data when tumor number was >6, higher values were truncated at six. A continuous variable as a potential risk factor was tested further for linearity before inclusion in the CRC model to identify whether transformations were needed. The linearity was checked by a four-knot restricted cubic spline model at Harrell's default percentiles (i.e., 5, 35, 65, and 95th) combined with a Wald-type test (29, 30).

Model performance, discrimination, and calibration were measured by Harrell's concordance-index (C-index), the time-dependent area under receiver operating characteristic curve (AUROC), and a calibration curve, respectively, in both the training and testing cohorts (31). The CRC model was compared with the seven well-recognized models [four-and seven criteria (4), six-and-12 score (5), HAP score (6), mHAP score (8), mHAP-II score (9), mHAP-III score (32), and ALBI grade (7)]. All models were subjected to 1,000- bootstrap resampling validation to calculate a relatively corrected C-index.

All statistical analyses were performed by using R version 3.5.1 (R Foundation for Statistical Computing, Vienna, Austria) with packages *survival, glmnet, rms, timeROC, caret, Hmisc*, and *compareC*. Statistical significance was set at *P* < 0.05 unless otherwise specified. *P*-values were two-sided.

## Results

### Patient Outcomes

Clinical characteristics were comparable between the training and testing cohorts (Table 1). Median OS was 19 (95% confidence interval (CI), 17.1–24.0) months in the training cohort and 21.8 (95% CI, 18.9–30.9) months in the validation cohort (log-rank test, *P* = 0.122). OS was censored in nine and 15 patients, respectively. The median survival was 19.9 (95% CI, 18.2–24.0) months in all patients, with 1-, 2-, and 3-years overall survival rate of 70.8, 40.1, and 26.0%, respectively. The median follow-up period was 66.2 ± 29.6 months (range 9.8–112.1 months). There was no significant survival difference among the five centers (log-rank test, *P* = 0.12).

**Table 1:** Characteristics of patients in the training and validation cohorts.

**Characteristics**	**Median (IQR)/Number (%)**			***P-*value**
	**Entire cohort**	**Training cohort**	**Validation cohort**	
	**(*N* = 162)**	**(*N* = 108)**	**(*N* = 54)**	
Age(year)	58 (47–66.8)	59 (47.2–72.5)	58 (47–66)	0.474
**Sex**				
Male	136 (83.9)	91 (84.3)	45 (83.3)	1
Female	26 (16.1)	17 (15.7)	9 (16.7)	
**Etiology**				0.076
HBV	109 (67.3)	78 (72.2)	31 (57.4)	
Others	53 (32.7)	30 (27.8)	23 (42.6)	
Tumor size (cm)	7.5 (4.4–10.2)	7.5 (4.2–11.1)	7.4 (4.5–10)	0.736
**Tumor Number**				
1	95 (58.7)	58 (53.6)	37 (68.5)	0.259
2	18 (11.1)	14 (13.0)	4 (7.4)	
3	13 (8.0)	11 (10.2)	2 (3.7)	
>3	36 (22.2)	25 (23.2)	11 (20.4)	
**BCLC stage**				0.775
A	8 (4.9)	5 (4.6)	3 (5.6)	
B	154 (95.1)	103 (95.4)	51 (94.4)	
**ALBI grade**				0.894
A	65 (40.1)	42 (38.9)	23 (42.6)	
B	93 (57.4)	63 (58.3)	30 (55.6)	
C	4 (2.5)	3 (2.8)	1 (1.8)	
**Child-Pugh class**				1
A	140 (86.4)	93 (86.1)	47 (87.0)	
B	22 (13.6)	15 (13.9)	7 (13.0)	
**AFP (ng/ml)**				0.127
<400	64 (39.5)	38 (35.2)	26 (48.2)	
≥400	98 (60.5)	70 (64.8)	28 (51.8)	
**AST (U/L)**	47.8 (31–70.5)	44.5 (34.1–68.6)	49.5 (30.8–75.2)	0.568
**ALT (U/L)**	39 (26.2–59)	34.5 (24.2–56.4)	41 (27–59)	0.552
**Prothrombin time (s)**	12.5 (11.7–13.9)	12.2 (11.8–13.3)	12.6 (11.7–14)	0.337
**Albumin (g/L)**	39 (35.8–43)	39.1 (35–41.9)	39 (36–43.4)	0.534
**Total bilirubin (μmol/L)**	19.6 (12.4–22.9)	15.9 (10.3–21.6)	19.9 (13.2–25.2)	0.094

*IQR, interquartile range; HBV, hepatitis B virus; BCLC, Barcelona Clinic Liver Cancer; ABLI, albumin-bilirubin; AFP, alpha-fetoprotein; AST, aspartate transaminase; ALT, alanine transaminase*.

*Median (IQR) are shown for continuous variables, whereas numbers (%) are shown for categorical variables*.

*P-values were calculated by the Mann-Whitney U-test for the continuous variables and the Fisher exact test for the categorical variables*.

### Construction of Radiomics Signature

**Figure 2:**
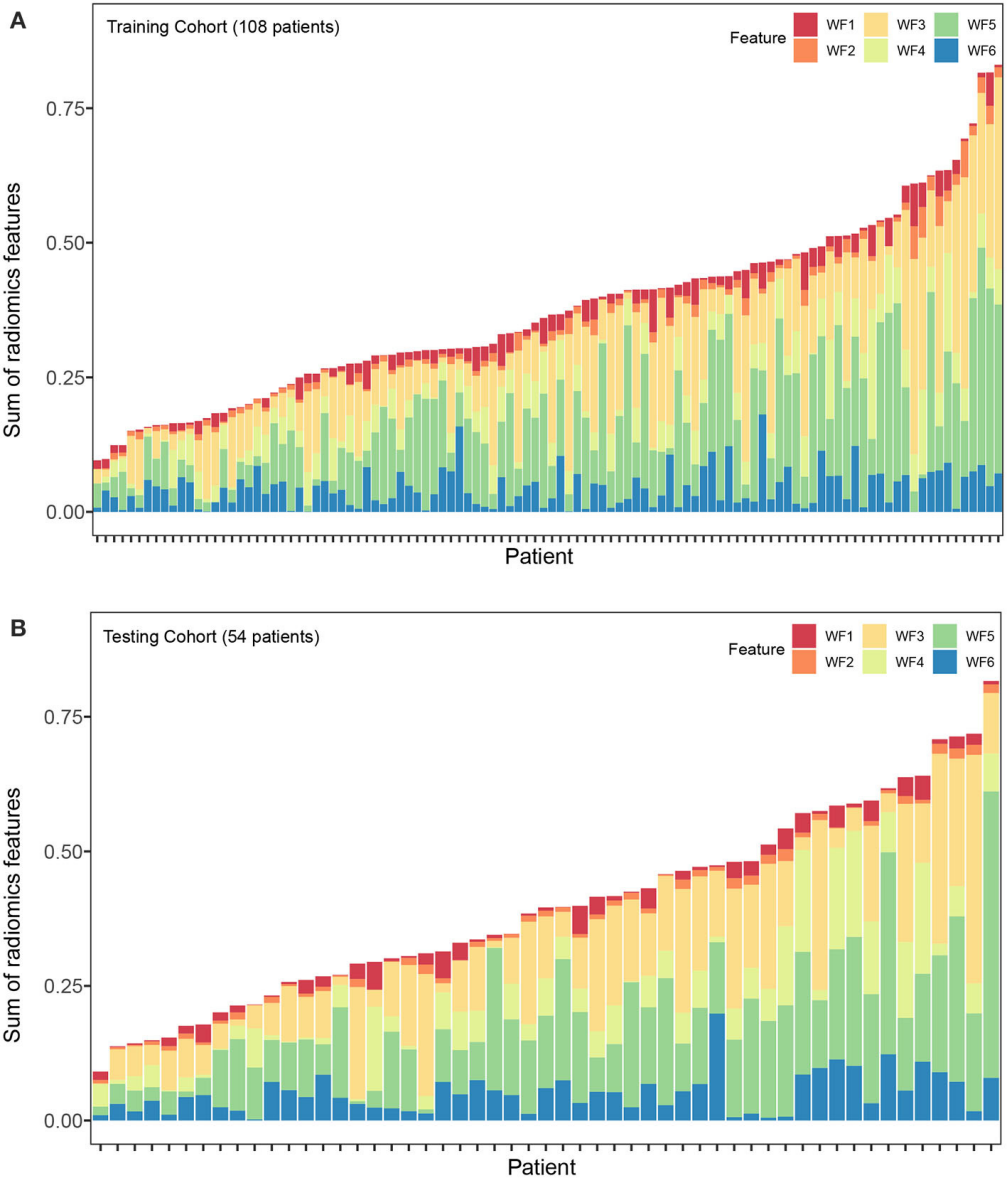
Stacked bars of the five selected features. **(A)** Training cohort (*n* = 108). **(B)** validation cohort (*n* = 54). Stacked bars of the selected features patient by patient. The height of each bar equal to the value of each feature multiply by the absolute value of its coefficient in the LASSO regression. From the stacked bars, it is convenient to visualize each component of the Rad-signature. LASSO, least absolute shrinkage and selection operator. F1, F2, F3, F4, F5, and F6 are corresponding to IDMN, Correlation, IMC1, SRLGLE, and LRLGLE in Table 2.

Altogether, 4,288 out of 5,152 features were reproducible following inter-observer ICC analysis (Figure S1). Further reduction of pair-wise correlations led to 1,393 independent features. Finally, six radiomics features with non-zero coefficients were selected after LASSO Cox regression from the training cohort (Figure S2). Of the six features, two were based on arterial phase imaging from tumor VOI and peritumoral VOI, separately, and the remaining four features were from tumor VOI on portal venous phase imaging. These radiomics features are detailed in Table 2. Figure 2 visualized each component's contribution to the Rad-signature; the stacked bars representing the six radiomics features were plotted for each patient.

**Table 2:** Features selected for predicting OS from CT images (*N* = 108).

**Feature No**.	**Imaging modality**	**VOI of feature extraction**	**Filter type**	**Feature class**	**Statistic**	Coefficients^*^
F1	Portal venous phase	Tumor	Wavelet_LLL	GLCM	IMC1	−0.1487
F2	Portal venous phase	Tumor	Wavelet_LLL	GLCM	IMC2	−0.0177
F3	Portal venous phase	Tumor	Wavelet_HLL	GLRLM	SRLGLE	−0.0282
F4	Arterial phase	Tumor	Wavelet_LHL	GLRLM	SRLGLE	−0.0600
F5	Arterial phase	Peritumoral region	Log.sigma.1.0.mm	GLDM	DNN	−0.1651
F6	Arterial phase	Peritumoral region	Wavelet_LHL	GLSZM	GLNN	−0.0571

*OS, Overall survival; VOI, Volume of interest; GLCM, Gray Level Co-occurrence Matrix; GLRLM, Gray Level Run Length Matrix; GLCM, Gray Level Co-occurrence Matrix; GLDM, Gray Level Dependence Matrix; GLSZM, Gray Level Size Zone Matrix. IMC, Informational Measure of Correlation; SRLGLE, Short_Run_Low_Gray_Level_Emphasis; DNN, Dependence Non-Uniformity Normalized; GLNN, Gray Level Non-Uniformity Normalized*.

* *Coefficients were derived from the LASSO Cox regression. Formula of the radiomics signature was as follows: radiomics signature = IMC1 × −0.1487 + IMC2 × -0.0177 + SRLGLE × −0.0282 + SRLGLE × −0.0600 +DNN × −0.1651 + GLNN × −0.0571*.

### The Combined Radiomics-Clinic Model Development and Validation

**Figure 3:**
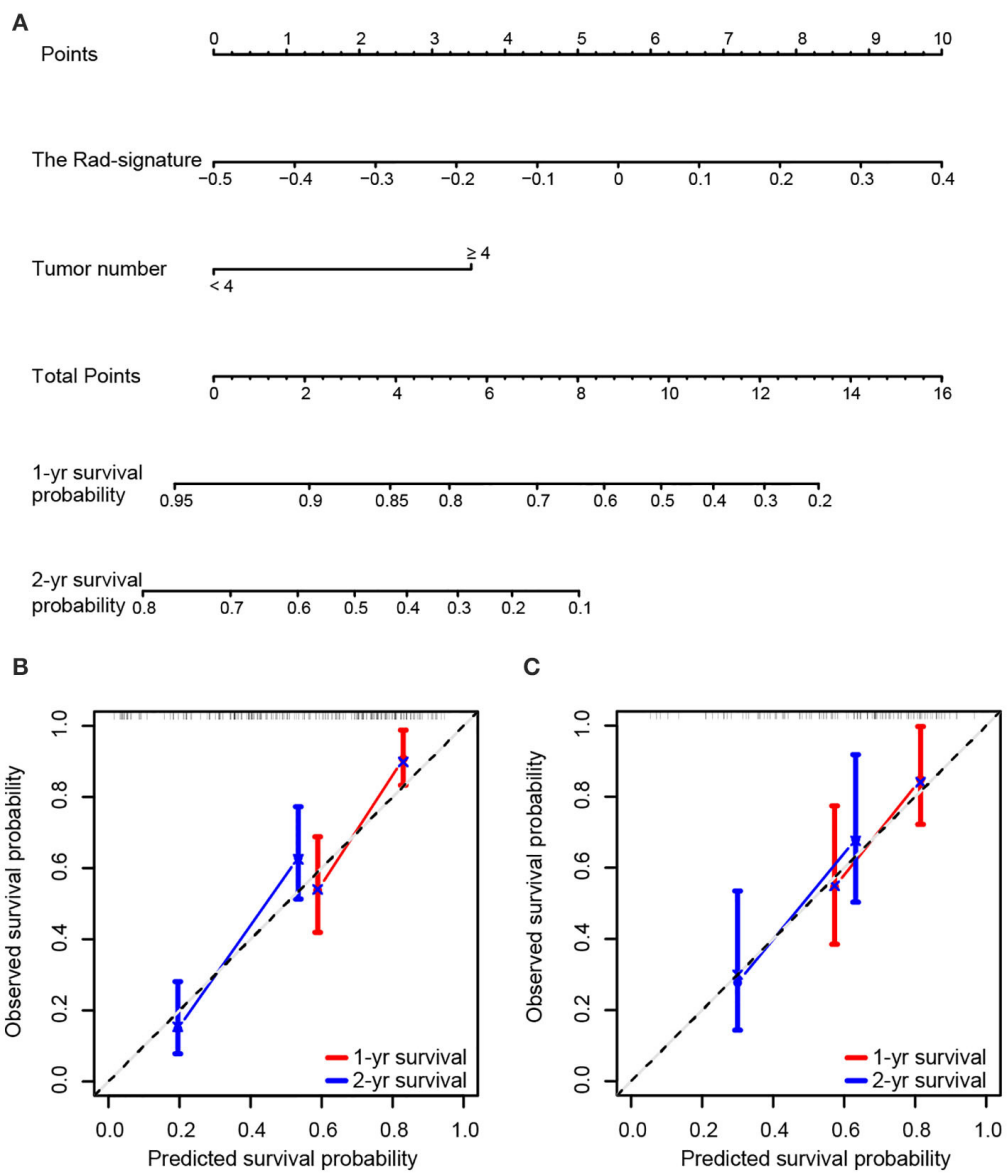
Nomogram and calibration curves of the combined radiomics-clinic (CRC) model. **(A)** Nomogram for 1- and 2-years survival probability based on the CRC model. Usage: Locate the patient's Rad-signature on the Rad-signature axis. Draw a line straight upward to the Points axis to determine how many points the patient arrived. Repeat the process for each variable. The points achieved for each of the variables were summed. Locate the sum on the Total Points axis. Draw a line straight down and find the patient's 1- or 2-years survival probability. Calibration curve of the CRC model for predicting 1- and 2-years survival in the training cohort **(B)** and testing cohort **(C)**. Model-predicted probability of overall survival is plotted on the x-axis; observed overall survival is plotted on the y-axis. The 45° line represents perfect prediction.

In the analyses, tumor size, AFP, and tumor number significantly predicted OS (*P* < 0.1). With multivariate analyses, continuous variables of tumor number and the Rad-signature were identified as independent prognostic factors (Table S2) and were analyzed further with restrictive cubic spline function to test linearity (Figure S3). The results showed that the effect of the Rad-signature was linear (non-linear *P*-values were 0.664 and 0.669 in the training and testing cohorts, respectively), but the tumor number was not (non-linear *P*-values were 0.059 and 0.016 in the training and testing cohorts, respectively). Therefore, only the Rad-signature could be treated as a continuous linear variable. For the convenience of clinical practice, tumor number was a categorized variable rather than a continuous variable with restrictive cubic spline transformation. To determine the optimal cutoff dichotomizing tumor number, we attempted all possible values by multivariate Cox regression analyses in both the training and testing cohorts. Results showed the models performed best in both the training and testing cohorts with a tumor number cut-off at four (Figure S3). The CRC model was finally established with tumor number (<4 vs. ≥4) and the Rad-signature (continuous). A nomogram for individualized prediction of 1- and 2-years survival probability was built based on the CRC model (Figure 3). The calibration curves of the CRC model in the training and testing cohorts were presented in Figure 3.

### Performance Comparison

**Figure 4:**
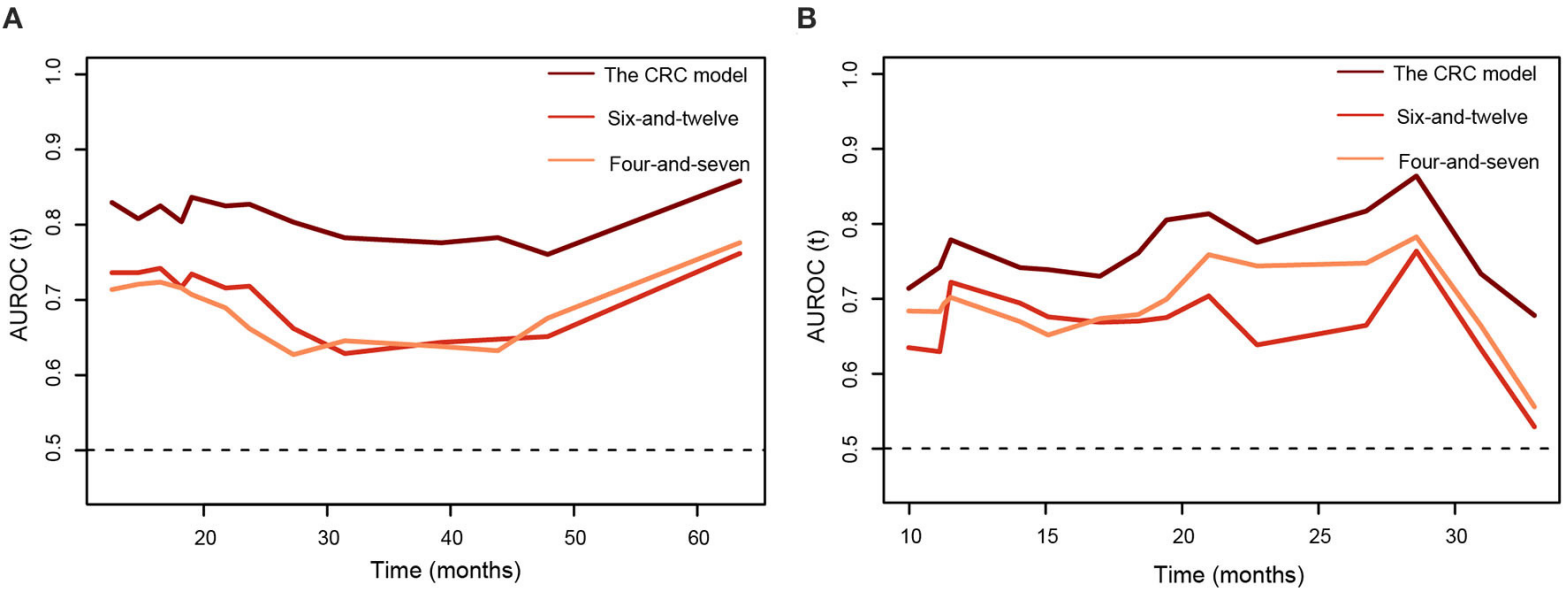
The time-dependent areas under receiver operating characteristic curves of the combined radiomics-clinic models, the six-and-twelve score, and the four-and-seven criteria for overall survival prediction. **(A)** training cohort. **(B)** testing cohort.

Table 3 summarized C-indices of the prognostic models. The CRC model showed a favorable performance, with C-indices of 0.73 [95% CI 0.68–0.79] and 0.70 [95% CI 0.62–0.82] in the training and testing cohort, respectively. Among the seven models, the six-and-12 score and four-and-seven criteria performed better than the other models, with C-indices of 0.64 [95% CI 0.58–0.70] and 0.65 [95% CI 0.55–0.75], respectively, in the testing cohort. Generally, time-dependent AUROC values of the CRC model were higher than both the six-and-12 score and four-and-seven criteria in the training and testing cohorts (Figure 4).

**Table 3:** Performance of models for overall survival.

**Model name**	**Predictors involved**	**C-index (95% CI)**
**Training Cohort**		
Rad-signature	Six radiomics features	0.68 (0.62–0.74)
CRC model	Tumor number (<4/≥4), Rad-signature	0.73 (0.68–0.79)
Six-and-twelve	Sum of tumor size and number	0.64 (0.58–0.70)
Four-and-seven	Within four tumors and 7 cm (yes/no), Child-pugh class A/B	0.63 (0.58–0.68)
HAP	Albumin (≥36 g/dl/ <36 g/dl), AFP (≤ 400 ng/ml/> 400 ng/ml), bilirubin (≤ 17 μmol/l/ >17 μmol/l), tumor size (≤ 7 cm/>7 cm)	0.55 (0.50–0.61)
mHAP	All predictors involved in HAP score but bilirubin	0.59 (0.53–0.65)
mHAP-II	All predictors involved in HAP score plus tumor number (1 /≥2)	0.57 (0.52–0.63)
mHAP-III	Albumin, AFP, bilirubin, tumor size, and tumor number	0.54 (0.46–0.60)
ALBI grade	Albumin, bilirubin	0.52 (0.45–0.56)
**Testing Cohort**		
Rad-signature	Five radiomics features	0.67 (0.56–0.79)
CRC model	Tumor number (<4/≥4), Rad-signature	0.70 (0.62–0.82)
Six-and-twelve	Sum of tumor size and number	0.64 (0.52–0.74)
Four-and-seven	Within four tumors and 7 cm (yes/no), Child-pugh class A/B	0.65 (0.55–0.75)
HAP	Albumin (≥36 g/dl/ <36 g/dl), AFP (≤ 400 ng/ml/>400 ng/ml), bilirubin (≤ 17 μmol/l/ >17 μmol/l), tumor size (≤ 7 cm/>7 cm)	0.55 (0.46–0.64)
mHAP	All predictors involved in HAP score but bilirubin	0.59 (0.47–0.71)
m-HAP-II	All predictors of the HAP score plus tumor number (1 /≥2)	0.61 (0.50–0.73)
mHAP-III score	Albumin, AFP, bilirubin, tumor size, and tumor number	0.58 (0.47–0.71)
ALBI grade	Albumin, bilirubin	0.56 (0.46–0.67)

*C-index, Concordance index; CI, confidence interval; Rad-signature, radiomics signature; AFP, alpha-fetoprotein; CRC, combined radiomics-clinic; HAP, hepatoma arterial-embolization prognostic; ABLI, albumin-bilirubin*.

### Survival Stratification

For the convenience of clinical practice, an individualized risk score was generated by a linear combination of the Rad-score and tumor number (<4 vs. ≥4) weighted by their respective coefficients from the multivariate Cox regression model. According to the median risk score (−0.0214) from the training cohort, patients were divided into two strata: stratum 1, a risk score <-0.0214., and stratum 2, the risk score >-0.0214.

**Figure 5:**
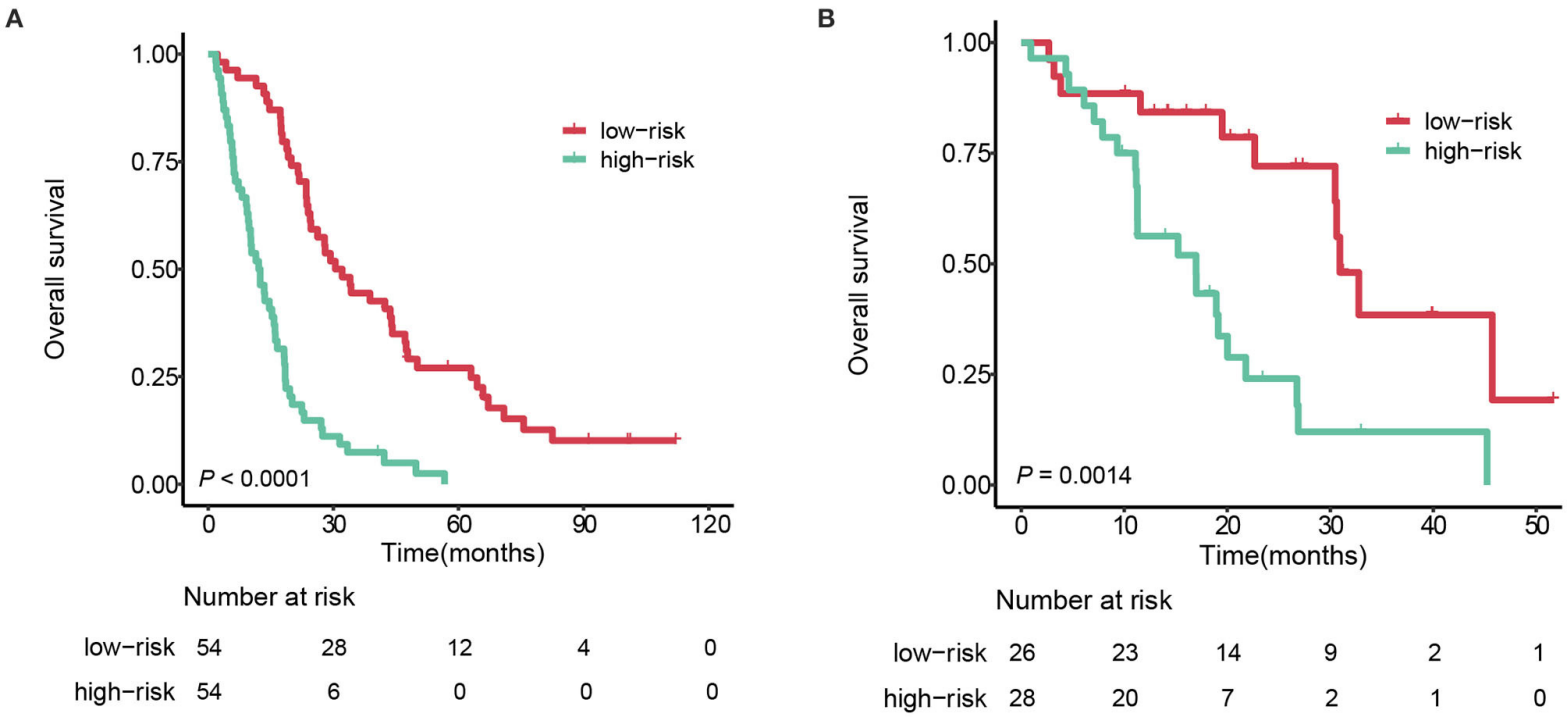
Kaplan-Meier survival curves of the 2 strata patients. **(A)** training cohort. **(B)** testing cohort.

In the training cohort, stratum 1 patients (median survival: 31.3 months [95%CI 24.5–4.1]) survived significantly longer than the stratum 2 patients (median survival: 12.5 months [95%CI 9.6–16.1]), with a hazard ratio 3.63 (95% CI 2.36–5.60, log-rank test *P* < 0.0001). Applying the same cutoff to the testing cohort, the median survivals of stratum 1 and 2 were 30.9 months (95%CI 30.5–NA) and 17.0 months (95%CI 11.3–26.8), respectively, with a hazard ratio 2.43 (95%CI 1.91–4.98, *P* = 0.0014). The survival curves of the two strata are plotted in Figure 5.

### Subgroup Analysis Based on Different Institutions

Data obtained from different institutions may be considered a potential confounder. The effects of different institutions on prognostic performance was investigated in the entire cohort. Following a bootstrap resampling procedure (1,000 bootstrap resamples), the C-indices of the radiomics signature in different subgroups ranged from 0.60 to 0.78 (Table S3). Consistently, Cox regression analyses applied in each center showed that the radiomics signature significantly analyzed survival (Table S3).

## Discussion

Patients with HCC receiving TACE have various clinical outcomes. In this study, we developed and independently validated a radiomics signature comprised of six radiomics features. The radiomics signature and tumor number (<4 vs. ≥4) were incorporated into a CRC model predicting OS in patients with HCC undergoing TACE. In comparison, seven previous well-recognized models were validated in our population, and the CRC model performed well-against the other models. Our study developed an accurate prognostic model, which would help identify the best candidates for TACE. This multicenter study included imaging data from different machines and CT scanning protocols in order to ensure the generalizability of the proposed model.

Our study identified that the radiomics signature comprising quantitative features was an independent prognostic factor for survival in patients with HCC undergoing TACE. Prognostic parameters from previous studies primarily measured tumor burden and liver function, seldom quantifying spatial heterogeneity within tumors, essential and neglected information correlated with HCC prognosis. Our study combined a novel radiomics approach with routinely used CT imaging to predict prognosis for patients with HCC receiving TACE. CT is regularly used in clinical practice to evaluate tumor burden and contains high-dimension minable data reflecting tumor heterogeneity (11). Both the arterial phase and portal venous phase images were investigated in this study and the results showed that radiomics features from portal venous phase images are also a critical component of the radiomics signature.

Radiomics analysis on arterial phase image was useful for prognosis prediction. This may be explained by that tumor texture patterns in arterial phase imaging could reflect tumor vascularization patterns, which was helpful for prognosis prediction (33). There may be two reasons explaining the importance of radiomics features from the portal venous images. One is that radiomics analysis of portal venous phase image was more useful for MVI prediction, which is a significant prognostic factor of HCC, than arterial phase images (34). The other is that texture of individual tumors in portal venous phase image can be heterogeneous and analysis of this heterogeneity has prognostic value (21). However, previous studies utilized only arterial phase CT imaging to investigate the capabilities of CT radiomics features to predict the treatment outcomes of HCC patients (20). The strength of radiomics analyses based on multiphasic enhancement images may be that multiphasic enhancement images can provide more comprehensive information on prognosis than single-phase images, while it also needs carefully segment tumor on each phase. Interestingly, the proposed radiomics signature included two peritumoral radiomics features from arterial phase imaging rather than the portal venous phase image. This finding was consistent with previous studies, in which the presence of peritumoral enhancement in arterial phase images indicated tumor biological aggressiveness (22, 35). Unlike previous studies, in which a peritumoral expansion distance of 1, 3, or 5 mm was set (21, 22), we selected a radial distance of 10 mm in this study. According to the guideline of pathological sampling of HCC specimens, liver tissue within a 10 mm distance was defined as the adjacent peritumoral region (36). The chances of microvascular invasion are high in this region, and therefore, 10 mm may represent a better peritumoral region correlated with prognosis evaluation (37).

When we applied the seven existing models to this population, the six-and-12 score and four-and-seven criteria performed better than the other five models. This result may be due to the exclusion of patients with vascular invasion, a significant negative factor in HCC prognosis from the target populations of the six-and-12 score, four-and-seven criteria studies, and our study (16). Conversely, the ALBI grade presented the worst performance when validated in this population, probably because this population preserved liver function, and various survival outcomes mainly resulted from tumor heterogeneity. The results of this study are largely consistent with the study that developed the six-and-12 score, and highlight the increasing importance of characterizing intratumor heterogeneity (5).

The study developing the six-and-12 score possessed the most similar patient population, in terms of ethnicity, HCC etiology, and BCLC stage distribution, with this current study. Correspondently, we found similar C-indices of the six-and-twelve score in our population and in the original study developing the six-and-12 score (5). The six-and-12 score presented as the sum of tumor size and tumor number; the CRC model included the rad-signature and tumor number (<4 vs. ≥4). The CRC model performed better than the six-and-12 score. This improvement may be mainly because the Rad-signature was established with high-dimensional whole-tumor radiomics features that measure the intensity and spatial textural heterogeneity of tumor image. The six-and-12 score included the tumor number as a continuous variable, which leads to counting every tumor. Conversely, tumor number was included as a dichotomized variable in the CRC model, and the cutoff is consistent with most staging algorithms such as the BCLC and Milan criteria (2). AFP was not included in the CRC model, but the prognostics ability of AFP level requires further analysis and validation in a large cohort study.

The retrospective nature of our study was the first of several limitations. Further evaluations in extensive prospective studies are needed to validate the results. Second, tumor VOI only included the single largest indexed lesion. Previous studies have validated the feasibility of assessing the largest lesion in survival analysis after TACE (38, 39), primarily because the largest lesion reflects the most aggressive behavior of HCC. Furthermore, manual delineation of tumor VOI can be time-consuming, limiting the model as an easy-to-use tool. With ongoing technological improvements of computer-aided algorithms, the tumor segmentation procedure, and feature screening could be designed as an automated workflow streamlined by computers and compatible with diagnostic radiology in standard clinical practice. Finally, while overall survival might be confounded by post-TACE variables, these variables were not involved in this study because they could not be used prior to the first TACE procedure. To reduce such biases, we included only treatment-naïve patients with well-preserved liver function in this population.

In conclusion, our study demonstrated the Rad-signature as an independent imaging predictor of survival in HCC patients undergoing TACE. For patients with BCLC B stage HCC or unresectable BCLC A stage HCC, the CRC model may prove valuable for the accurate prediction of OS and selection of best candidates for TACE.

## Data Availability Statement

The original contributions presented in the study are included in the article/Supplementary Material, further inquiries can be directed to the corresponding author/s.

## Ethics Statement

The studies involving human participants were reviewed and approved by the Ethics Review Committee of the Zhongda Hospital. Written informed consent for participation was not required for this study in accordance with the national legislation and the institutional requirements.

## Author Contributions

X-PM, Y-CW, C-QL, and SJ: conception and design. X-PM, Y-CW, and C-QL: development of methodology. X-PM, B-YZ, C-FN, JX, JJ, and X-MZ: acquisition of data. X-PM, Y-CW, C-QL, B-YZ, QY, ZZ, and GY: analysis and interpretation of data (e.g., statistical analysis, computational analysis). SJ take final responsibility for this article. All authors: writing, revision, read, and final approval of the manuscript.

## Conflict of Interest

The authors declare that the research was conducted in the absence of any commercial or financial relationships that could be construed as a potential conflict of interest.

## Abbreviations

CI: confidence interval
C-index: Concordance index
CRC: combined radiomics-clinic
CT: computed tomography
HAP: hepatoma arterial-embolization prognostic
HCC: hepatocellular carcinoma
LASSO: the least absolute shrinkage and selection
OS: overall survival
Rad-signature: radiomics signature
TACE: transarterial chemoembolization
VOI: volume of interest.
