# Supplementary material for: Radiomics Analysis on Multiphase Contrast-Enhanced CT: A Survival Prediction Tool in Patients With Hepatocellular Carcinoma Undergoing Transarterial Chemoembolization
Source: Front Oncol. 2020 Jul 21;10:1196. doi: 10.3389/fonc.2020.01196 (PMC7396545; doi:10.3389/fonc.2020.01196)
Supplement: Supplementary file 1 [file Data_Sheet_1.pdf]

Supplementary Material

1 Appendix E1–E2

Appendix E1

Tumor segmentation guideline

Tumor VOIs were segmented manually layer-by-layer on the contrast-enhanced arterial and portal venous phases CT images by the two radiologists who were blinded to the clinical outcomes. For tumors with fuzzy or unclear boundaries, especially in the arterial phase, the readers referred to portal venous phase images and carefully delineated ROI after the discussion of the two readers. In the case of multifocal disease, the largest tumor was selected as the lesion of interest. The largest tumor was the target lesion (or one of the target lesions) treated during initial TACE. Reader 1 finished all the tumor segmentations. The final segmentation results of tumor VOI were validated by a senior radiologist with 15-year abdominal imaging experience.

Peritumoral volume of interest (VOI)

In entire cohort of 162 CT scans, the pixel sizes ranged from 0.54 × 0.54 mm to 0.84 × 0.84 mm with a median size of 0.68 × 0.68 mm (see Table S1). The peritumoral VOI was automatically reconstructed layer-by-layer according to the entire tumor VOI contours with a dilation algorithm. With a fixed dilated distance of 10 mm, by considering the individual pixel size of each scan, the number of pixels dilated in each layer for each peritumoral VOI for were calculated as follows: 10/ pixel size (in mm).

To make sure the whole peritumoral VOI was always been restricted in the liver parenchyma region, only the intersected VOI between the dilated region and whole liver region was kept during the dilation procedure. The three-dimensional (3D) whole liver was automatically segmented using a convolutional neural network model, which is trained using CT image data from the Liver Tumor Segmentation Challenge (https://competitions.codalab.org/competitions/15595). After the automatic liver segmentations were finished, all segmentation results were visually checked, and unsatisfactory segmentations were manually adjusted by reader 1.

Appendix E2

Imaging preprocessing

First, images were isotropically resampled to 1× 1× 1 mm^3 voxels using Bspline interpolation, which maintained equal distances between neighboring voxels in all directions. Interpolation to isotropic voxel makes texture feature sets to be rotationally invariant and allows comparison among image data from different samples and centers (1). Second, intensities discretization inside the image VOI was done. The range of gray level intensities inside the VOI was –1,055 to 845; with a fixed bin width 25HU, a new bin was assigned for every intensity. This procedure was used to make the

calculation of texture features tractable (2). The two procedures were performed on both tumor VOIs and peritumoral VOIs.

Detailed methods of radiomics feature extraction

For each type of VOI, radiomics feature set consisted of four categories:

Category one: 3D-Shape-based features (14 features)

The shape features include Voxel Volume, Maximum3D Diameter, Mesh Volume, Major Axis Length, Sphericity, Least Axis Length, Elongation, Surface Volume Ratio, Maximum2D Diameter Slice, Flatness, Surface Area, Minor Axis Length, Maximum2D Diameter Column, and Maximum2D Diameter Row

Category two: The First Order features (18 features)

Interquartile Range, Skewness, Kurtosis, Uniformity, Median, Energy, Robust Mean Absolute Deviation, Mean Absolute Deviation, Total Energy, Maximum, Root Mean Squared, 90Percentile, Minimum, Entropy, Range, Variance, 10Percentile, Mean

Category three: the second-order features, Gray Level Cooccurence Matrix (22 features)

Joint Average, Joint Entropy, Cluster Shade, Maximum Probability, Inverse Difference Moment Normalized (Idmn), Joint Energy, Contrast, Difference Entropy, Inverse Variance, Difference Variance, Inverse Difference Normalized (Idn), Inverse Difference Moment (Idm), Correlation, Autocorrelation, Sum Entropy, Sum Squares, Cluster Prominence, Informational Measure of Correlation (Imc) 2, Imc1, Difference Average, Inverse Difference (Id), Cluster Tendency

Category three: the second-order features, Gray Level Run Length Matrix (16 features)

Long Run High Gray Level Emphasis, Short Run Low Gray Level Emphasis, Gray Level Variance, Low Gray Level Run Emphasis, Gray Level Non-Uniformity Normalized, Run Variance, Gray Level Non-Uniformity, Long Run Emphasis, Short Run High Gray Level Emphasis, Run Length Non-Uniformity, Short Run Emphasis, Run Percentage, Long Run Low Gray Level Emphasis, Run Entropy, High Gray Level Run Emphasis, Run Length Non-Uniformity Normalized

Category three: the second-order features, Gray Level Size Zone Matrix (16 features)

Gray Level Variance, Zone Variance, Gray Level Non-Uniformity Normalized, Size Zone Non-Uniformity Normalized, Size Zone Non-Uniformity, Gray Level Non-Uniformity, Large Area Emphasis, Small Area High Gray Level Emphasis, Zone Percentage, Large Area Low Gray Level Emphasis, Large Area High Gray Level Emphasis, High Gray Level Zone Emphasis, Small Area Emphasis, Low Gray Level Zone Emphasis, Zone Entropy, Small Area Low Gray Level Emphasis

Category three: the second-order features, Neighbouring Gray Tone Difference Matrix (5 features)

Coarseness, Complexity, Contrast, Busyness, Strength

Category three: the second-order features, Gray Level Dependence Matrix (14 features)

Large Dependence High Gray Level Emphasis, Gray Level Variance, High Gray Level Emphasis, Dependence Entropy, Dependence Non-Uniformity, Gray Level Non-Uniformity, Small Dependence Emphasis, Small Dependence High Gray Level Emphasis, Dependence Non-Uniformity Normalized, Large Dependence Emphasis, Large Dependence Low Gray Level Emphasis, Dependence Variance, Small Dependence Low Gray Level Emphasis, Low Gray Level Emphasis.

Category four: high-order filters features

Features in category four are extracted from filtered image sets; the aforementioned18 first-order features and 73 second-order features were extracted from the following filtered image sets.

LoG: Laplacian of Gaussian filter, edge enhancement filter. A Laplacian of Gaussian image is obtained by convolving the image with the second derivative (Laplacian) of a Gaussian kernel. The width of the filter (mm) in the Gaussian kernel is determined by sigma. A low sigma emphasis on fine textures (change over a short distance), where a high sigma value emphasizes coarse textures (gray level change over a large distance). Five sigma values were used in this study (1 mm, 2 mm, 3 mm, 4 mm, and 5 mm) and thus five types of filtered images were generated.

Names of features extracted from Laplacian of Gaussian filtered images were prefixed with LoG. sigma_1. 0, LoG. sigma_2. 0, LoG. sigma_3. 0, LoG. sigma_4. 0, or LoG. sigma_5. 0.

Wavelet: Wavelet filtering, yields 8 decompositions per level (all possible combinations of applying either a High or a Low pass filter in each of the three dimensions), and thus eight types of filtered images were generated.

Names of features extracted from wavelet filtered images were prefixed with Wavelet_HHH, Wavelet_HHL, Wavelet_HLL, Wavelet_HLH, Wavelet_LHH, Wavelet_LLL, Wavelet_LLH, or Wavelet_LHL.

The calculation formula and definition of each feature are shown at https://pyradiomics. readthedocs. io/en/latest/ .

Reference:

1. Zwanenburg A, Leger, S., Vallières, M., and Löck, S. Image biomarker standardisation initiative feature definitions. *arXiv:161207003v7 [csCV]* (2018).
2. Yip SS, Aerts HJ. Applications and limitations of radiomics. *Phys Med Biol* (2016) 61:R150-66. doi: 10.1088/0031-9155/61/13/R150.

2 Supplementary Figures and Tables

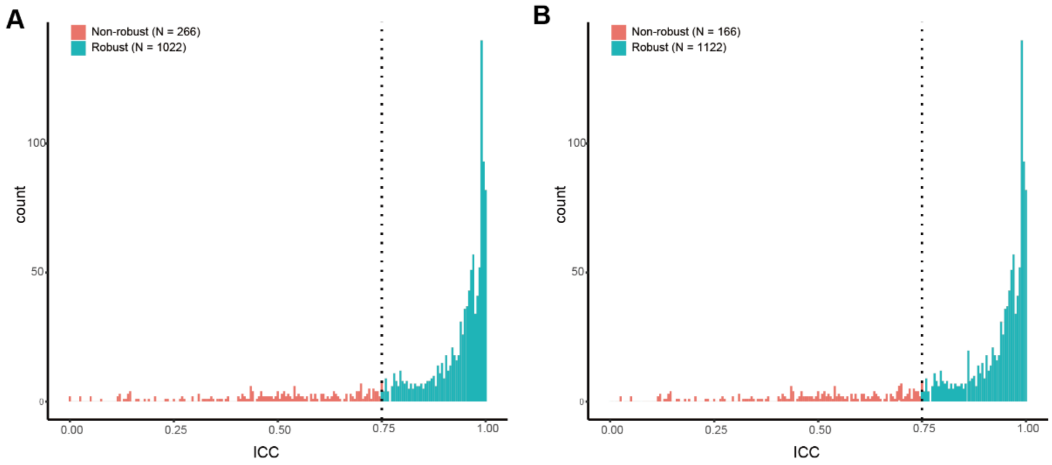

Figure S1. Histogram of the interclass correlation coefficient (ICC) for radiomics features. (A) For intratumoral radiomics features from arterial phase, 1,022 radiomics features were robust. (B) For intratumoral radiomics features from portal venous phase, 1,122 radiomics features were robust. A total of 2,144 features from intratumoral region are robust, correspondently, 2,144 features from peritumoral regions are considered as robust. Thus, a total of 4,288 radiomics features were selected.

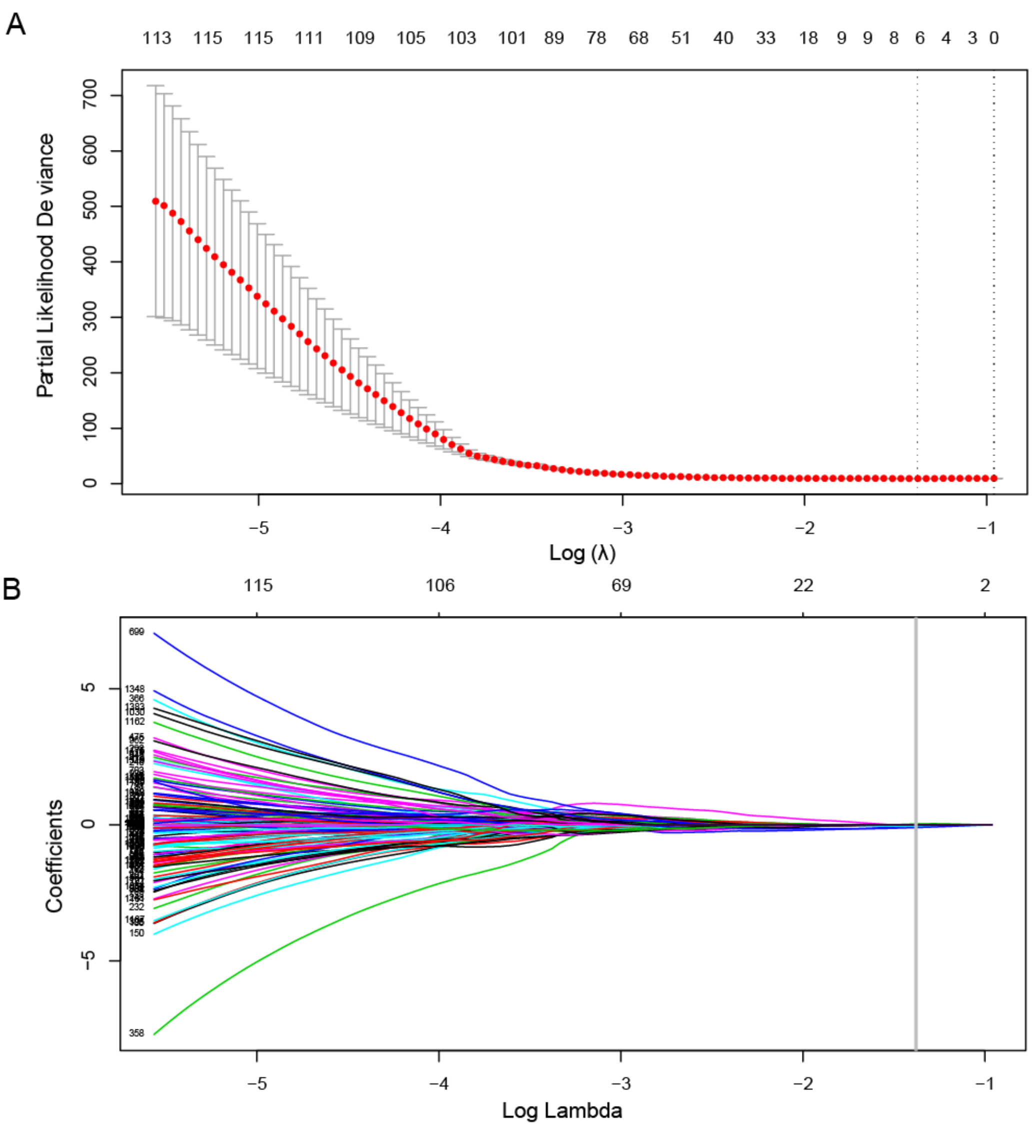

Figure S2. Turning parameter (λ) selection in the least absolute shrinkage and selection operator (LASSO) model. (A) Ten-time cross-validation for λ selection. Solid vertical lines represent partial likelihood deviance standard error (SE). The dotted vertical lines are located at the optimal values by minimum criteria and 1-SE criteria. We plotted the partial likelihood deviance versus log (λ), in which λ is the tuning parameter. In this study, λ was chosen via minimum criteria. A value λ = 0.2518531, with log (λ) = –1.378909. (B) LASSO coefficient profiles of the features. A coefficient plot was generated against the log (λ) sequence. The vertical line was located at the value selected where the minimum λ led to five non-zero coefficients.

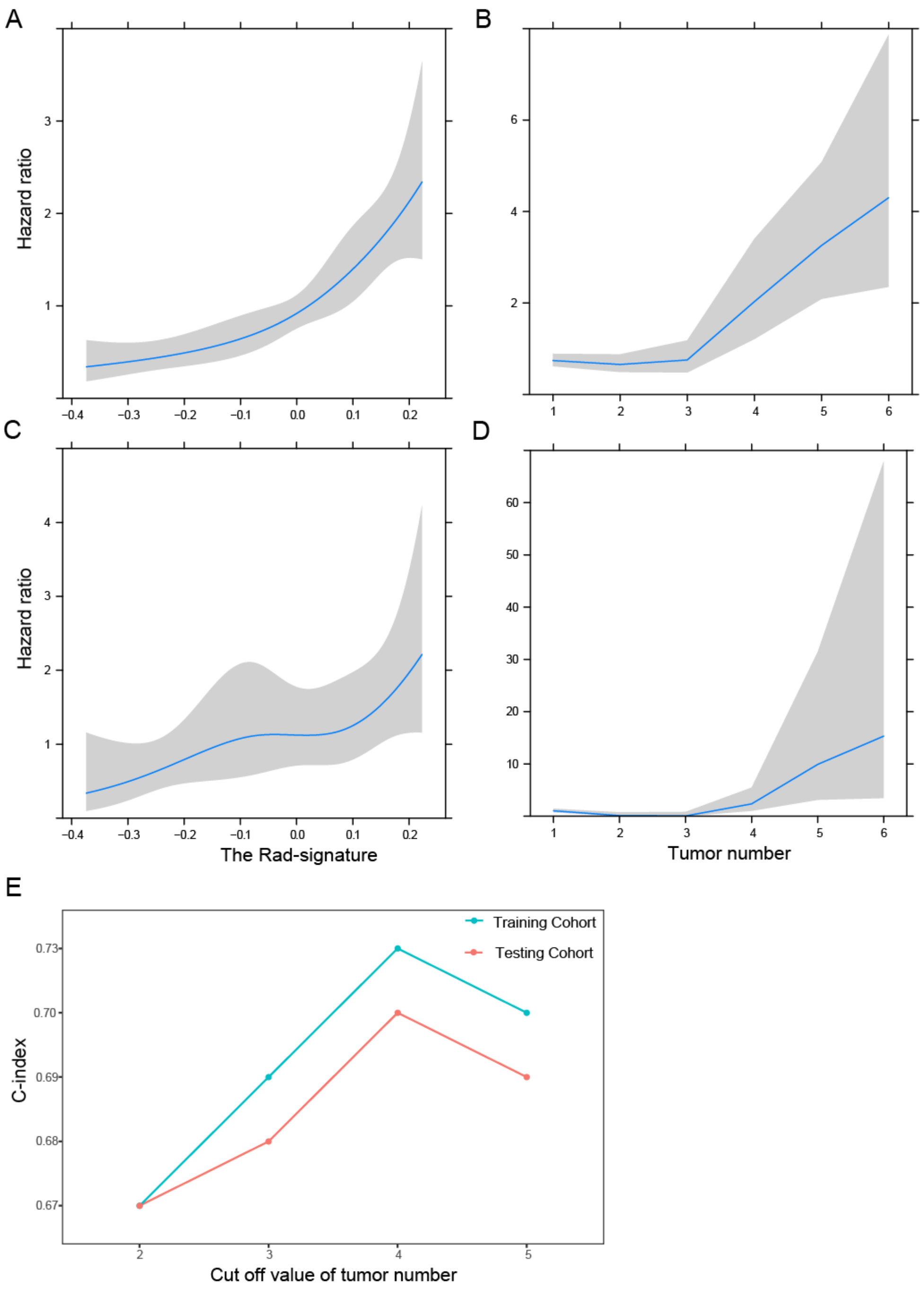

Figure S3. Restricted cubic spline analyses and identification of the optimal cutoff for tumor number. Relation between the Rad-signature (4-knot restricted cubic spline transformation) and hazard ratios (95% CI) adjusted by tumor number in training cohort (A) and testing cohort (C), Relation between tumor number (4-knot restricted cubic spline transformation) and hazard ratios (95%CI) adjusted by the Rad-signature in training cohort (B) and testing cohort (D). (E) C-indices of

the combined radiomics-clinic (CRC) model according to different cutoff values of tumor number in the training and testing cohort.

Table S1. Technical data of CT images in each center

Variable	All Centers (n = 162)	Center No. 1 (n = 35)	Center No. 2 (n = 43)	Center No. 3 (n = 20)	Center No. 4 (n = 42)	Center No. 5 (n = 22)
**Slice Thickness* (mm)**	5 (1.25–10)	5 (3–5)	6 (1.25–8)	5 (1.25–5)	8 (6–10)	5 (5–5)
**Pixel Spacing* (mm)**	0.68 (0.55–0.84)	0.68 (0.58–0.778)	0.70 (0.59–0.78)	0.70 (0.59–0.84)	0.66 (0.55–0.81)	0.68 (0.61–0.76)

NOTE. *data are median and range.

Table S2. **Cox regression analyses of prognostic factors in the training cohort**

	Univariate analysis		Multivariate analysis	
**Variable**	**HR (95%CI)**	***P* value**	**HR (95%CI)**	***P* value**
**Age (year)**	0.79 (0.51–1.2)	0.298		
**Sex* (Male vs. Female)**	1.02 (0.6–1.7)	0.949		
**Child-Pugh class* (A vs. B)**	1.09 (0.66–1.8)	0.738		
**HBV* (no/yes)**	1 (0.64–1.6)	1		
**Tumor size (cm)**	1.05 (1.00–1.10)	0.056	0.93 (0.87–1.45)	0.060
**Tumor number**	1.34 (1.1–1.6)	<0.001	1.27 (1.12–1.45)	<0.0001
**AFP* (≥400 ng/ml/<400 ng/ml)**	1.79 (1.2–2.8)	<0.001	1.42 (0.90–2.44)	0.122
**AST (U/L)**	1 (1–1)	0.231		
**ALT (U/L)**	0.998 (0.99–1)	0.295		
**Prothrombin time (sec)**	1.05 (0.96–1.2)	0.285		
**Albumin (g/L)**	0.972 (0.94–1)	0.127		
**TBIL (μmol/L)**	1 (0.98–1)	0.942		
**The Rad-signature**	7.33 (3.62–14.82)	<0.001	6.86 (2.84–16.57)	<0.0001

NOTE. * The forepart of the parentheses was set as the reference group in univariate analyses. HR, hazard ratio; CI, confidence interval; HBV, hepatitis B virus; AFP, α-fetoprotein; AST, aspartate transaminase; ALT, alanine transaminase; TBIL, total bilirubin. The Rad-signature, radiomics signature.

Table S3. Subgroup analysis of the radiomics signature for survival prediction in the entire cohort

Variable	Center No. 1 (n = 35)	Center No. 2 (n = 43)	Center No. 3 (n = 20)	Center No. 4 (n = 42)	Center No. 5 (n = 22)
**C-index (95% CI)**	0.61 (0.50–0.73)	0.63 (0.50–0.77)	0.60 (0.39–0.79)	0.66 (0.56–0.76)	0.78 (0.69–0.87)
P* Value	0.043	0.008	0.040	0.019	< 0 .001

NOTE. CI, confidence interval; C-index were calculated after a bootstrap resampling procedure (1,000 bootstrap resamples); * *P* value was obtained by Cox regression analyses for association of the radiomics signature with overall survival in each center.
